# Methylome and transcriptome profiles in three yak tissues revealed that DNA methylation and the transcription factor ZGPAT co-regulate milk production

**DOI:** 10.1186/s12864-020-07151-3

**Published:** 2020-10-20

**Authors:** Jinwei Xin, Zhixin Chai, Chengfu Zhang, Qiang Zhang, Yong Zhu, Hanwen Cao, Cidan Yangji, Xiaoying Chen, Hui Jiang, Jincheng Zhong, Qiumei Ji

**Affiliations:** 1 State Key Laboratory of Hulless Barley and Yak Germplasm Resources and Genetic Improvement, Lhasa, Tibet China; 2 Institute of Animal Science and Veterinary, Tibet Academy of Agricultural and Animal Husbandry Sciences, Lhasa, Tibet China; 3 Key Laboratory of Qinghai-Tibetan Plateau Animal Genetic Resource Reservation and Utilization, Sichuan Province and Ministry of Education, Southwest Minzu University, Chengdu, Sichuan China

**Keywords:** Milk production, DNA methylation, Transcription factor, Epigenetic regulation

## Abstract

**Background:**

Domestic yaks play an indispensable role in sustaining the livelihood of Tibetans and other ethnic groups on the Qinghai-Tibetan Plateau (QTP), by providing milk and meat. They have evolved numerous physiological adaptations to high-altitude environment, including strong blood oxygen transportation capabilities and high metabolism. The roles of DNA methylation and gene expression in milk production and high-altitudes adaptation need further exploration.

**Results:**

We performed genome-wide DNA methylome and transcriptome analyses of breast, lung, and biceps brachii muscle tissues from yaks of different ages. We identified 432,350 differentially methylated regions (DMRs) across the age groups within each tissue. The post-mature breast tissue had considerably more differentially methylated regions (155,957) than that from the three younger age groups. Hypomethylated genes with high expression levels might regulate milk production by influencing protein processing in the endoplasmic reticulum. According to weighted gene correlation network analysis, the “hub” gene ZGPAT was highly expressed in the post-mature breast tissue, indicating that it potentially regulates the transcription of 280 genes that influence protein synthesis, processing, and secretion. The tissue network analysis indicated that high expression of HIF1A regulates energy metabolism in the lung.

**Conclusions:**

This study provides a basis for understanding the epigenetic mechanisms underlying milk production in yaks, and the results offer insight to breeding programs aimed at improving milk production.

**Supplementary information:**

**Supplementary information** accompanies this paper at 10.1186/s12864-020-07151-3.

## Background

Domestic yaks play an indispensable role in sustaining the livelihood of Tibetans and other ethnic groups on the Qinghai-Tibetan Plateau (QTP), in the Himalayas, and in the connecting Central Asian highlands. They provide milk, meat, hides, fiber, fuel, and transportation [1, 2]. Yak milk is not only an important source of high-quality protein, especially containing high quantities of essential amino acids, but also rich in immunoglobulins, antimicrobial peptides and growth factors [3, 4]. Protein content and composition are essential for the transformation of milk into cheese and other milk-derived products, and therefore important for the dairy industry. Previous researches indicated that manipulation of DNA transcription and posttranscriptional regulation can improve the efficiency of milk production. The synthesis of fat, protein, and lactose in milk can be regulated by the activity of specific transcription factors, non-coding RNAs, and alterations of the chromatin structure in mammary epithelial cells [5]. Despite such advancements in research, little is currently known about the physiological and cellular regulation required for milk protein synthesis and secretion in yak. We hypothesized that the genes responsible for milk production were regulated by DNA methylation and that distinct sub-modules of correlated expression variation could be identified. In this study, we performed genome-wide DNA methylome and transcriptome analyses of yak lung, breast, and biceps brachii muscle tissues at four different stages of development to identify the regulatory networks associated with milk protein synthesis, metabolism, and secretion.

## Results

### Global DNA methylation and gene expression in the breast, lungs, and biceps brachii muscle at different ages

**Fig. 1:**
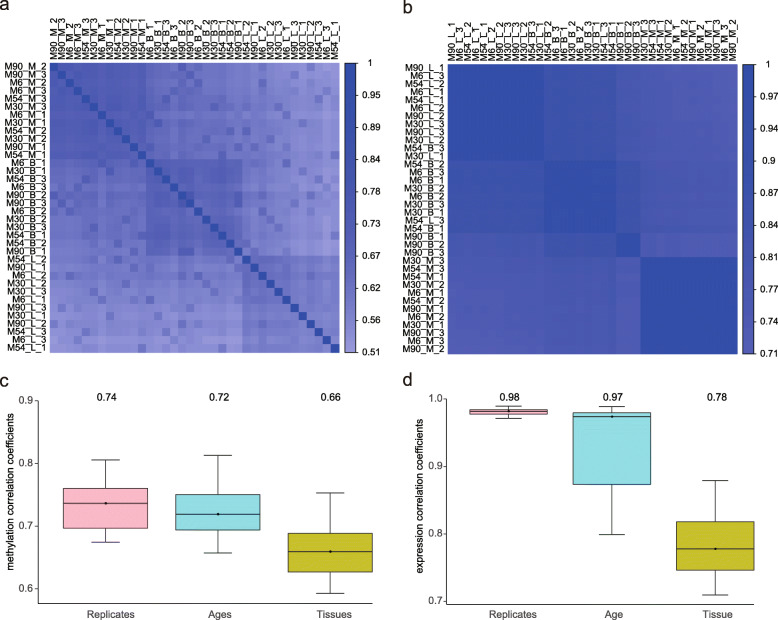
Global DNA methylation and gene expression among samples. Pearson’s correlation analysis based on the methylation of CpG sites (**a**) and gene expression (**b**) among samples. Boxplot of Pearson’s correlation coefficients between replicates, ages, or tissues for methylation (**c)** and gene expression (**d**). M6, M30, M54 and M90 represent different 6, 30, 54 and 90 months old, respectively. B, L, and M represent breast, lung, and biceps brachii muscle, respectively. 1, 2, and 3 represent different replicates

We generated the methylomes and transcriptomes of lung, breast, and biceps brachii muscle tissues from 12 female Riwoqe yaks at four different stages (*n* = 3/stage) of development: 6 months old (MO) (young), 30 MO (pre-mature), 54 MO (mature) and 90 MO (post mature). Among these, only the post-mature yaks (90 months) were lactation, with ~ 3.16 kg/day milk yield. After performing sequence quality control and filtering, we obtained a single-base resolution methylome covering 85.6% (27,471,373/32,092,725) of CpG sites across the genome with an average depth of 22.5×. We first calculated pairwise Pearson’s correlations of CpG sites with at least 10× coverage depth across all samples, which were well clustered by tissue types (Fig. 1a). The correlation of CpG methylation levels for biological replicates was strong (median Pearson’s *r* = 0.74), the correlation of CpG methylation levels between ages (median Pearson’s *r* = 0.72) was relatively weaker and the correlation between tissues was weakest (median Pearson’s *r* = 0.66) (Fig. 1c). We aligned the transcriptome sequencing data for all samples to our newly assembled yak genome reference, and we subsequently obtained the transcripts. In total, we obtained 20,504 transcripts, that were then annotated to the Gene Ontology (GO) [6], InterPro [7], Kyoto Encyclopedia of Genes and Genomes (KEGG) [8], Swiss-Prot [9], and TrEMBL [10] databases (Table S1). We also calculated pairwise Pearson’s correlations of all transcripts and obtained similar results to those of DNA methylation (Fig. 1b). Biological replicates showed the highest correlation coefficients, while different tissues showed the lowest correlation coefficients (Fig. 1d).

### Differentially methylated regions among the age groups

**Fig. 2:**
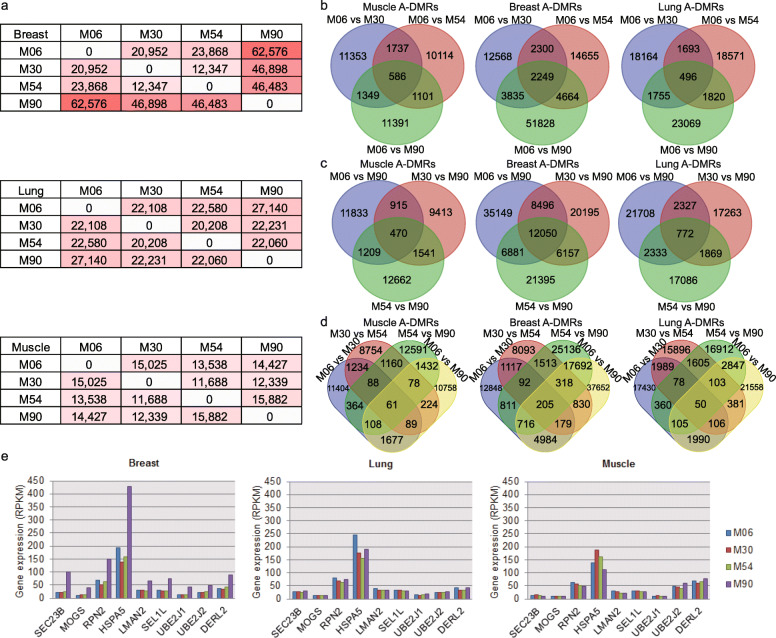
Overview of age-associated DMRs. **a** Basic statistics for A-DMRs within each tissue. **b** Expression levels of 9 enriched genes in “protein processing in endoplasmic reticulum (ER)”. Overlap of A-DMRs associated with the 6-month group (**c**), 90-month group (**d**), and 30- and 54- months groups (**e**) in the muscle, breast, and lung respectively

We determined differentially methylated regions (DMRs) across age groups within the breast, lung, and biceps brachii muscle tissues (Table S2, S3, S4). Within the lung and biceps brachii muscle tissues, age groups did not differ in age-related DMRs (A-DMRs), but post-mature breast tissue had considerably more DMRs (155,957) than the three younger age groups (Fig. 2a). We then investigated the correlations between DMRs and their corresponding differentially expressed genes. The ratio of negatively to positively correlated gene pairs was 1.02 for promoters with DMRs and 0.95 for gene bodies with DMRs. Not every methylation was correlated with the expression of its associated gene, due to the gene regulation complexity [11].

At ~ 90 months, ~ 120 days after giving birth for the third time, yaks are in the lactation period (milk yield of ~ 3.16 kg/day), so it is possible that the observed methylation partially controls yak lactation. Since promoter methylation decreases gene expression [11], we selected 375 hypomethylated promoter genes (highly expressed) along with 207 hypermethylated promoter genes (lowly expressed) from post-mature yak breast tissues. The hypomethylated (highly expressed) genes were only enriched in “protein processing in endoplasmic reticulum (ER)” (9 genes, 2.964-fold enrichment, *p* = 0.0049). Specifically, the genes are involved in vesicle trafficking (SEC23B), oligosaccharide linking (MOGS, RPN2), folding and assembly (HSPA5), transportation (LMAN2, SEL1L), and ubiquitination and degradation (UBE2J1, UBE2J2, DERL2) [12, 13]. These genes were significantly upregulated at 90 months in breast tissues, but not in lung and biceps brachii muscle tissues (Fig. 2b). Based on this data, methylation might help regulate milk production by influencing protein processing in the endoplasmic reticulum during the lactation period.

We also examined A-DMRs that overlapped across age groups. Young and post-mature tissues rarely shared A-DMRs when comparing the lung and muscle tissues (for young tissues, muscle: 586 A-DMRs, breast: 2249 A-DMRs, lung: 496 A-DMRs; for post-mature tissues, muscle: 470 A-DMRs, breast: 12,050 A-DMRs, lung: 772 A-DMRs) (Fig. 2c, d). Pre-mature and mature stages also rarely shared A-DMRs across muscle and lung tissues (Fig. 2e), suggesting that methylation patterns were already established at the young stage and that no extensively divergent epigenetic difference occurred across different age groups under natural high-altitude conditions.

### Consensus network analysis for tissues and age groups

**Fig. 3:**
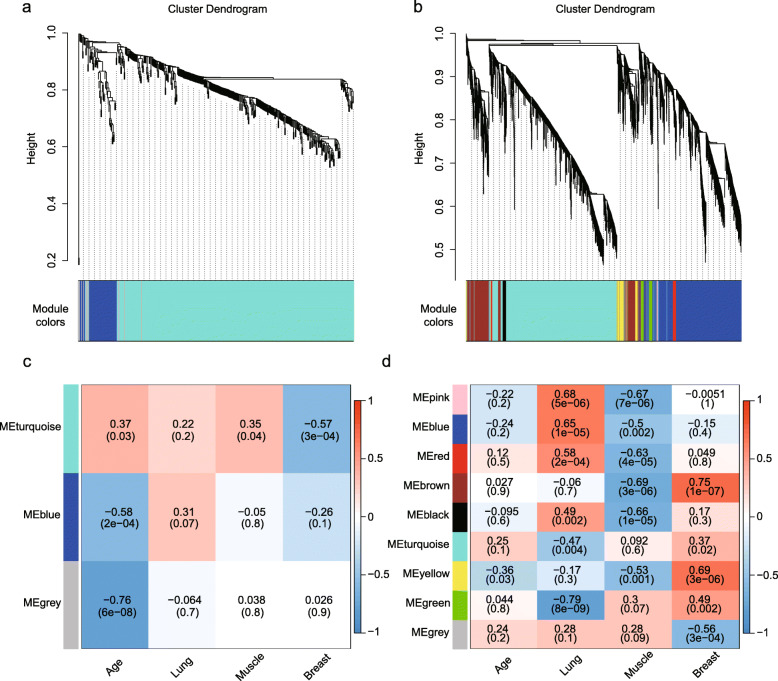
Modules of consensus networks and correlation with traits. Consensus networks from the age (**a**) or tissue (**b**) curves. Gene expression similarity was determined using a pair-wise weighted correlation metric and clustered according to a topological overlap metric into modules; assigned modules are colored on the bottom, and gray genes were not assigned to any module. Consensus network modules for age (**c**) and tissue (**d**) correlated with traits using the eigenmodule (the first principal component of the module). The correlation coefficients and the p-value in parenthesis are provided underneath; color-coding refers to the correlation coefficient (legend at right)

We first performed a multi-way ANOVA test for each gene across all samples (*n* = 36) to test the null hypothesis that the gene expression level did not differ among age groups and tissues. At the threshold for significance (*p* < 0.05), 417 age-related and 8560 tissue-related genes were selected for further weighted gene correlation network analysis (WGCNA), which uses network topography to group genes into modules based on correlations [14]. Next, we conducted WGCNA for tissue- and age-related gene expression respectively to identify a “consensus network”–a common pattern of genes that are correlated in all conditions. We performed a consensus network, module statistic, and eigengene network analyses to identify modules, to assess relationships between modules and traits, and to study the relationships between co-expression modules [15]. The consensus networks identified for tissues and age groups had clearly delineated modules (Fig. 3a, b), and the modules identified were significantly correlated with tissues and age groups (Fig. 3c, d).

### Age network analysis indicates that ZGPAT might regulate milk production

**Fig. 4:**
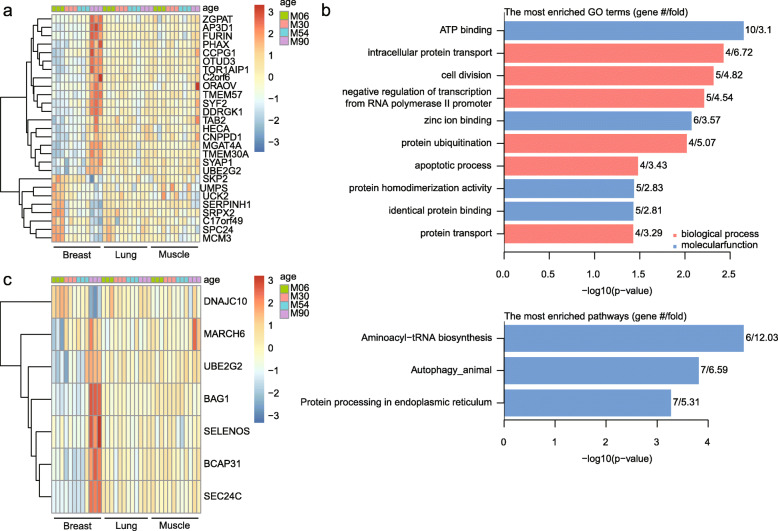
“Hub” genes and potential target genes of ZGPAT in the age network. **a** Expression level of 27 “hub” genes. **b** Enrichment analysis of ZGPAT’s potential target genes. **c** Expression level of 7 genes in “protein processing in endoplasmic reticulum”, which was enriched from potential target genes of ZGPAT

Within the age-related gene network, the largest module (“turquoise”, *n* = 356) had negative correlation for breast tissue (*r* = − 0.57, *p* = 3e-04) and age (*r* = 0.37, *p* = 0.03) and a positive correlation for biceps brachii muscle tissues (Table S5). The breast tissue had a stronger signal than that of age and may have overwhelmed the signal from age. Genes in this module were enriched in the GO categories of “protein polyubiquitination,” “RNA polymerase II core promoter proximal region sequence-specific DNA binding,” “ATP binding,” “transcription, DNA-templated,” and “negative regulation of transcription from RNA polymerase II promoter” (*p*-values of 0.000344, 0.000822, 0.000991, 0.00139, and 0.00244, respectively). The “blue” (*n* = 48) and “grey” (*n* = 13) modules showed only a negative correlation with age (*r* = − 0.58, *p* = 2e-04; *r* = − 0.76, *p* = 6e-08, respectively) and exhibited no enrichment of GO categories for genes. After applying the threshold of the absolute value of gene significance for age (|GS| > 0.5) and module membership measures (|MM| > 0.6) in each module, we defined 20 and 7 “hub” genes in the “turquoise” and “blue” modules (Table 1). The gene expression of the “hub” genes was well clustered by modules, which was consistent with the negative correlation with age (“turquoise” *r* = 0.37, “blue” *r* = − 0.58). The upregulated expression level of the “hubs” in breast tissue at 90 months old indicated that the “turquoise” module had a stronger correlation with breast tissue than with age (Fig. 4a).

**Table 1:** List of “hub” genes in the consensus network for age

Yak ID	Gene symbol	Module color	Gene significance	*p*-value	Module membership	*p*-value
BmuPB009868	AP3D1	turquoise	0.513965075	0.001344116	0.868945999	6.37E-12
BmuPB004083	TMEM30A	turquoise	0.505722627	0.001652657	0.848054843	6.65E-11
BmuPB000726	DDRGK1	turquoise	0.53596122	0.000754414	0.842070133	1.22E-10
BmuPB019011	OTUD3	turquoise	0.543916749	0.000606215	0.828680521	4.37E-10
BmuPB000091	CNPPD1	turquoise	0.51195228	0.001414359	0.814537409	1.50E-09
BmuPB006815	TOR1AIP1	turquoise	0.544705745	0.000593032	0.809789221	2.21E-09
BmuPB007137	MGAT4A	turquoise	0.545969144	0.000572452	0.798170686	5.51E-09
BmuPB007385	HECA	turquoise	0.603273223	9.84E-05	0.770629907	3.85E-08
BmuPB019443	SYAP1	turquoise	0.500377628	0.001884511	0.733120646	3.68E-07
BmuPB009825	PHAX	turquoise	0.561938984	0.00036184	0.70497442	1.59E-06
BmuPB008032	UBE2G2	turquoise	0.52385538	0.001041698	0.70208134	1.83E-06
BmuPB012517	SYF2	turquoise	0.568328068	0.000299194	0.699367268	2.08E-06
BmuPB001610	FURIN	turquoise	0.567906296	0.000303008	0.697046314	2.32E-06
BmuPB000679	ZGPAT	turquoise	0.504624296	0.001698141	0.683958391	4.25E-06
BmuPB007049	SKP2	turquoise	−0.527608908	0.000943732	− 0.669790419	7.91E-06
BmuPB000224	C2orf6	turquoise	0.531191165	0.000857925	0.652871802	1.59E-05
BmuPB007420	TAB2	turquoise	0.505905132	0.001645204	0.652844304	1.59E-05
BmuPB018569	ORAOV1	turquoise	0.51035386	0.001472421	0.637043276	2.95E-05
BmuPB010550	CCPG1	turquoise	0.644779605	2.19E-05	0.612874899	7.08E-05
BmuPB012521	TMEM57	turquoise	0.57317319	0.000258349	0.60967979	7.91E-05
BmuPB013324	MCM3	blue	−0.583585649	0.000186982	0.841505398	1.29E-10
BmuPB010064	SPC24	blue	−0.509962181	0.001486965	0.744497748	1.93E-07
BmuPB003102	C17orf49	blue	−0.526803776	0.000964031	0.741805787	2.26E-07
BmuPB015902	SERPINH1	blue	−0.607769763	8.44E-05	0.721017606	7.04E-07
BmuPB016582	SRPX2	blue	−0.51838468	0.001200558	0.698151166	2.20E-06
BmuPB012996	UCK2	blue	−0.588274454	0.000161067	0.677420455	5.68E-06
BmuPB015372	UMPS	blue	−0.503577413	0.001742514	0.648111658	1.92E-05

We used the AnimalTFDB 3.0 database [16] to examine transcription factors in these 27 “hubs” and found that ZGPAT encodes a transcription regulator protein and was significantly upregulated in breast tissue at 90 months of age (Fig. 4a). Previous study reported that this protein specifically binds the 5′-GGAG [GA] A [GA]A-3′ consensus sequence and represses transcription by recruiting the chromatin multi-complex NuRD to target promoters [17]. ZGPAT was highly expressed in breast tissue at 90 months, and it potentially regulated the transcription of 280 genes (weight > 0.15) in the network from the “turquoise” module. In order to identify the most important cellular activities controlled by this TF regulatory network, we analyzed over-represented GO biological process and molecular function terms, as well as KEGG pathways. These potential target genes were enriched in the GO categories of “protein binding,” “ATP binding,” and “zinc ion binding,” among others, and the KEGG categories of “aminoacyl-tRNA biosynthesis,” “autophagy animal,” and “protein processing in endoplasmic reticulum” (Fig. 4b). These enriched GO terms and KEGG pathways likely help regulate protein synthesis, processing, and secretion in breast tissue. For example, 6 of 7 genes from the “protein processing in endoplasmic reticulum” category were also upregulated at 90 months of age in breast tissue (Fig. 4c) and involved in multiple processes in the endoplasmic reticulum, including vesicle trafficking (SEC24C), folding and assembly (SELENOS), transportation (BCAP31), and ubiquitination and degradation (BAG1, UBE2G2, and MARCH6) [12, 13]. Only DNAJC10 was downregulated at 90 months of age in breast tissue. This gene encodes an endoplasmic reticulum co-chaperone that is part of the endoplasmic reticulum-associated degradation complex involved in recognizing and degrading misfolded proteins [13].

### Tissue network analysis indicates regulative role of HIF1A in lung

**Fig. 5:**
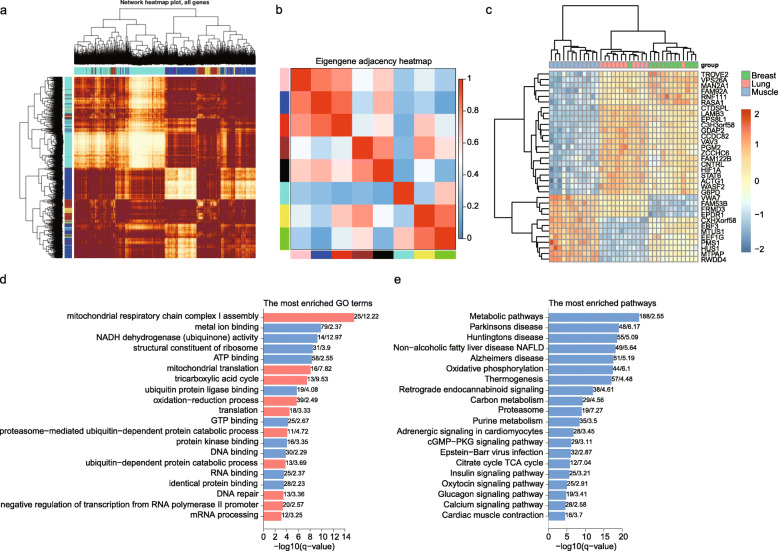
Modules and “hub” genes in the tissue network. **a** WGCNA modules of the tissue-related genes, (**b**) correlations between modules showed by the eigenmodule adjacency heatmap, (**c**) expression level of “hub” genes in the tissue network, enrichment analysis of GO (**d**) and KEGG (**e**) for potential target genes of HIF1A, and the number of enriched genes and enrichment fold are indicated on the right

Within the tissue-related gene module network, four modules showed a positive correlation and two showed a negative correlation with the lung; all significant module-trait relationships were negative in the muscle but positive in the breast (Fig. 3d). Moreover, 99.54% of the total 8560 tissue-related genes were related to the top 4 modules (“turquoise,” *n* = 3833; “blue,” *n* = 2795; “brown,” *n* = 1052; “yellow,” *n* = 339) (Fig. 5a, Table S6), and these modules were also highly correlated with other modules; for example, “brown,” “yellow,” and “black” showed a high eigengene adjacency with each other (Fig. 5b).

We applied the more stringent threshold absolute value of gene significance for the age and module membership measures in the top four modules to identify “hub” genes in the “turquoise,” “blue,” “brown,” and “yellow” modules. With the threshold values of |GS| > 0.7 and |MM| > 0.8, 34 “hub” genes were identified in the “turquoise” module. Twenty-four hubs were then filtered from the gene significance of module-lung relationships, and 10 were filtered from the gene significance of module-breast relationships (Table 2). These were further divided into 3 clusters by hierarchical clustering, which showed high expression levels in the breast (cluster 1), lung (cluster 2), and biceps brachii muscle (cluster 3) tissues, with distinct clustering patterns by tissue (Fig. 5c).

**Table 2:** List of “hub” genes in the consensus network for tissue

Yak ID	Gene symbol	Module color	Tissue	Gene significance	*p*-value	Module membership	*p*-value
BmuPB014336	EEF1G	turquoise	lung	−0.73907039	2.64E-07	0.826748126	5.20E-10
BmuPB017352	PMS1	turquoise	lung	−0.71599797	9.13E-07	0.850552958	5.12E-11
BmuPB000878	MTPAP	turquoise	lung	−0.715583465	9.33E-07	0.912877613	8.73E-15
BmuPB018762	CXHXorf58	turquoise	lung	−0.709433358	1.27E-06	0.82262518	7.50E-10
BmuPB014450	RWDD4	turquoise	lung	−0.708526806	1.33E-06	0.923713618	9.94E-16
BmuPB011005	HUS1	turquoise	lung	−0.707137304	1.43E-06	0.875052199	2.97E-12
BmuPB005540	MTUS1	turquoise	lung	−0.704482832	1.62E-06	0.871626441	4.58E-12
BmuPB011453	EBF3	turquoise	lung	−0.703373458	1.71E-06	0.870717052	5.13E-12
BmuPB010608	LAMB3	turquoise	lung	0.70783756	1.38E-06	−0.85067459	5.05E-11
BmuPB004299	C3H3orf58	turquoise	lung	0.708872549	1.31E-06	−0.88522675	7.61E-13
BmuPB020871	G6PD	turquoise	lung	0.708930411	1.30E-06	−0.90727231	2.41E-14
BmuPB007438	ZCCHC6	turquoise	lung	0.709740967	1.25E-06	−0.84738754	7.13E-11
BmuPB007592	CTDSPL	turquoise	lung	0.711634321	1.14E-06	−0.8813581	1.30E-12
BmuPB004894	HIF1A	turquoise	lung	0.713237417	1.05E-06	−0.87302984	3.84E-12
BmuPB020508	FAM122B	turquoise	lung	0.720139618	7.37E-07	−0.82428698	6.48E-10
BmuPB018102	CCDC82	turquoise	lung	0.720592854	7.20E-07	−0.90665968	2.68E-14
BmuPB014539	CNTRL	turquoise	lung	0.722130497	6.64E-07	−0.87149974	4.65E-12
BmuPB003882	VAV3	turquoise	lung	0.725601285	5.53E-07	−0.89496378	1.82E-13
BmuPB020153	EPS8L1	turquoise	lung	0.727495147	4.99E-07	−0.82286844	7.34E-10
BmuPB010308	PGM2	turquoise	lung	0.746811608	1.69E-07	−0.83795918	1.83E-10
BmuPB004008	GDAP2	turquoise	lung	0.754806874	1.05E-07	−0.88661303	6.26E-13
BmuPB012568	WASF2	turquoise	lung	0.757428944	8.92E-08	−0.83874427	1.69E-10
BmuPB011966	ACTG1	turquoise	lung	0.772114072	3.49E-08	−0.84373776	1.03E-10
BmuPB014173	STAT6	turquoise	lung	0.80896084	2.37E-09	−0.84981355	5.53E-11
BmuPB015106	MAN2A1	turquoise	breast	0.705124693	1.57E-06	−0.84782348	6.81E-11
BmuPB008614	VPS26A	turquoise	breast	0.731553581	4.01E-07	−0.88669366	6.18E-13
BmuPB018496	FAM92A	turquoise	breast	0.718223061	8.14E-07	−0.85598454	2.85E-11
BmuPB005078	RASA1	turquoise	breast	0.705902582	1.51E-06	−0.85869193	2.11E-11
BmuPB011476	FAM53B	turquoise	breast	−0.728187435	4.81E-07	0.848680696	6.23E-11
BmuPB008348	EPDR1	turquoise	breast	−0.710771064	1.19E-06	0.832481139	3.08E-10
BmuPB003453	TROVE2	turquoise	breast	0.72654448	5.26E-07	−0.80669368	2.84E-09
BmuPB021200	VWA7	turquoise	breast	−0.722814498	6.41E-07	0.814033145	1.56E-09
BmuPB010528	RNF111	turquoise	breast	0.737234077	2.92E-07	−0.80146106	4.28E-09
BmuPB015811	FRMD3	turquoise	breast	−0.739223421	2.61E-07	0.809147869	2.33E-09

According to the AmalTFDB 3.0 database [16], EBF3, HIF1A, and STAT6 were annotated as transcription factors. EBF3 encodes a member of the early B-cell factor (EBF) family of DNA binding transcription factors, and may function as a tumor suppressor in several types of cancer [18]. STAT6 is a member of the STAT family of transcription factors, which form homo- or heterodimers that translocate to the cell nucleus where they act as transcription activators [19]. HIF1A, hypoxia-inducible factor-1, functions as a master transcriptional regulator of the cellular and systemic homeostatic response to hypoxia [20]. HIF1A was upregulated in the lung to potentially control the transcription of 2008 genes (weight > 0.15), which were enriched in multiple GO biological process categories, molecular function categories and KEGG pathways. Most of the enriched GO terms and KEGG pathways were related to energy metabolism. Specifically, enriched GO terms included “mitochondrial respiratory chain complex I assembly,” “NADH dehydrogenase (ubiquinone) activity,” “ATP binding,” “mitochondrial translation,” “tricarboxylic acid cycle,” and “GTP binding” while enriched KEGG pathways included “thermogenesis,” “carbon metabolism,” and “citrate cycle TCA cycle” (Fig. 5d, e). Mitochondria function as the primary energy producers of the cell and serves as the hub for a variety of immune pathways as the center of biosynthesis, oxidative stress response, and cellular signaling [21]. NADH dehydrogenase is a core subunit of the mitochondrial membrane respiratory chain and is believed to contribute to the minimal assembly required for catalysis [22]. The protein which binds ATP or GTP, carries three phosphate groups esterified to a sugar moiety and provides energy and phosphate sources for the cell [23, 24]. The tricarboxylic acid cycle is a series of metabolic reactions in aerobic cellular respiration, and is the most important metabolic pathway for the energy supply to the body [25].. The “thermogenesis” pathway is essential for warm-blooded animals, because it ensures normal cellular and physiological functions under challenging environmental conditions [26].

## Discussion

Previous studies described transcription profiling of the mammary gland in livestock (including cattle [27], sheep [28], and goat [29]) and DNA methylation profiling of the mammary gland in cattle [30]. These studies showed temporal and spatial specificity in the methylome and transcriptome profiles of the mammary gland in different species, but they only reported the differential gene expression profile of the mammary gland; and the regulatory network is still unknown. In this study, we for the first time generated the methylomes and transcriptomes of lung, breast, and biceps brachii muscle tissues from yak at four different stages of development (6, 30, 54, and 90 months; young, pre-mature, mature, and post-mature, respectively). We found that breast tissue at 90 months showed considerably differential methylation levels compared with other month groups, but lung and biceps brachii muscle tissues did not. Enrichment analysis for upregulated genes with hypomethylated DMRs from breast tissues of 90-month-old yaks showed that DNA methylation might regulate the activation of the “protein processing in endoplasmic reticulum (ER)” pathway. Because only the 90-month-old yaks were in the lactation period, it appears that DNA methylation regulates milk production by influencing protein processing in the endoplasmic reticulum.

In this study, hub genes were identified by WGCNA. The data show that the hub genes with the highest MM and GS in modules of interest should be candidates for further research. This study identified turquoise module genes associated with milk yield, and 20 genes were considered hub genes, showing the highest mRNA expression level in breast tissue at 90 months when yaks enter the lactation period. In these hub genes, ZGPAT was annotated as a transcription factor that potentially regulated the transcription of 280 genes in the “turquoise” module network, which was enriched in the KEGG categories of “aminoacyl-tRNA biosynthesis,” “autophagy animal,” and “protein processing in endoplasmic reticulum”. This result suggests that ZGPAT helps with regulating protein synthesis, processing, and secretion in breast tissue. Moreover, the 7 genes potentially regulated by ZGPAT in “protein processing in endoplasmic reticulum” were totally different from the aforementioned 9 genes regulated by hypomethylation, illustrating that DNA methylation and transcription factor possibly co-regulate milk production. In addition, the tissue network analysis confirms the importance of HIF1A in regulating energy metabolism, which is necessary for adaptations to low temperature and hypoxia in high altitudes.

## Conclusions

The results of this comprehensive study provide a solid basis for understanding the roles of DNA methylation and transcriptional network underlying milk protein synthesis and high-altitude adaptation in yaks. This information advances our understanding of the regulatory network in the mammary gland at different developmental stages and may help inform breeding programs aimed at improving milk production.

## Methods

### Animals and samples

In total, twelve female yaks (belonging to an indigenous yak breed that lives at altitudes of 3800–4000 m above sea level in Riwoqe, Tibet, China) were collected between June and December of 2016 from private farms (Keqiong farm, Riwoqe, Tibet, China). They were grouped with 3 replicates into age categories of 6, 30, 54, and 90 months. At the time of slaughter, their mean live weights were 44.93 kg (6 months old), 153.06 kg (30 months old), 188.3 kg (54 months old), and 243.56 kg (90 months old). Only the 90 month-old yaks were lactating, producing ~ 3.16 kg/day milk yield (~ 120 days after giving birth for the third time). The 54-month-old yaks were in a dry period (~ 540 days after giving birth for the first time) [31]. The blood relation between the last three generations, which were housed simultaneously and fed the same diets, was unknown. The yaks were not fed the night before they were slaughtered and were humanely sacrificed with the following procedures: (1) showered with clean water close to body temperature (35–38 °C), (2) electrically stunned (120 V dc, 12 s) prior to exsanguination, (3) sacrificed while in the coma by bloodletting from carotid artery and jugular vein, (4) and dissected rapidly to obtain breast, lung, and biceps brachii muscle tissue samples, which were immediately frozen in liquid nitrogen, and stored at − 80 °C until RNA and DNA extraction.

### Whole genome bisulfite sequencing

The QIAamp DNA Mini Kit (Qiagen, Hilden, Germany) was used to isolate the high-quality DNA from each sample. According to the manufacturer’s instructions, 1 μg of genomic DNA was fragmented by sonication to a mean size of approximately 250 bp and subsequently used for whole genome bisulfite sequencing (WGBS) library construction using an Acegen Bisulfite-Seq Library Prep Kit (Acegen, Shenzhen, GD, China). Briefly, fragmented DNA was end-repaired, 5′-phosphorylated, 3′-dA-tailed, and then ligated to methylated adapters. The methylated adapter-ligated DNAs were purified using 1× Agencourt AMPure XP magnetic beads (Beckman Coulter, Brea, CA, USA) and subjected to bisulfite conversion with a ZYMO EZ DNA Methylation-Gold Kit (Zymo research, Irvine, CA, USA). The converted DNAs were then amplified using 25 μl HiFi HotStart U+ RM and 8-bp index primers with a final concentration of 1 μM each. The constructed WGBS libraries were then analyzed with an Agilent 2100 Bioanalyzer (Agilent Technologies, SantaClara, CA, USA), quantified with a Qubit fluorometer with Quant-iT dsDNA HS Assay Kit (Invitrogen, Carlsbad, CA,USA), and finally sequenced on an Illumina Hiseq X ten sequencer (PE150 mode) (Illumina, San Diego, CA, USA).

### Methylation calculation and identification of DMRs

Low quality reads that contained more than 5 ‘N’s or had a low-quality value for over 50% of the sequence (Phred score < 5) were filtered. The sequencing reads of the samples were aligned to the yak reference genome [32] using BSMAP (Version 2.74) [33]. The methylated CpG (mCG) sites were identified following a previously described algorithm [34]. The methylation levels for each sample were calculated using in-house Perl scripts. Differentially methylated regions (DMRs) were identified using metilene (Version 0.2–6) within a 500 bp sliding window at 250 bp steps with at least 10 CpGs covered by over 10× sequence reads, applying the thresholds of differential methylation β > =15%, FDR for two-dimensional Kolmogorov-Smirnov-Test *p*-value < 0.05 [35].. The enrichment analyses were conducted using WebGestalt (WEB-based Gene SeT AnaLysis Toolkit) [36].

### Total RNA extraction, library preparation, and sequencing

The TRIzol reagent (Invitrogen, Carlsbad, CA, USA) was used to isolate the total RNA of each sample. The purity, concentration, and integrity of RNA were checked using the NanoPhotometer spectrophotometer (IMPLEN, Westlake Village, CA, USA), the Qubit RNA Assay Kit in Qubit 2.0 Fluorometer (Life Technologies, Carlsbad, CA, USA), and the RNA Nano 6000 Assay Kit of the Bioanalyzer 2100 System (Agilent Technologies, SantaClara, CA, USA), respectively. For each sample, 3 μg high-quality RNA was used as input material for RNA-seq library preparation. First, ribosomal RNA was removed using the Epicentre Ribo-Zero rRNA Removal Kit (Epicentre, Madison, WI, USA). Next, the rRNA-depleted RNA was used to create sequencing libraries using the NEBNext Ultra Directional RNA Library Prep Kit for Illumina (NEB, Ipswich, MA, USA). Finally, the library products were purified using 1× Agencourt AMPure XP magnetic beads (Beckman Coulter, Brea, CA, USA) and the Agilent Bioanalyzer 2100 System (Agilent Technologies, SantaClara, CA, USA) was employed to assess the library quality. Clustering of the index-coded samples was completed on a cBot Cluster Generation System using the TruSeq PE Cluster Kit v3-cBot-HS (Illumina, San Diego, CA, USA), and then the libraries were sequenced on the Illumina HiSeq X Ten Platform to generate 150 bp paired-end reads.

### Quality analysis, transcriptome assembly, and abundance estimation

Clean reads were obtained by removing reads containing the adapter or poly-N and by removing low quality reads (over 10% of the sequence with a quality value < 30) from the raw data using in-house Perl scripts. All downstream analyses were based on the good-quality clean reads. Paired-end clean reads were mapped to the yak reference genome [32] with STAR (available at https://github.com/alexdobin/STAR/releases). The mapped reads of each sample were assembled using StringTie [37]. Next, all sample transcriptomes were merged to reconstruct a comprehensive transcriptome using Perl scripts. After the final transcriptome was generated, StringTie and edgeR were used to estimate the expression levels of all transcripts [38]. StringTie was used to assess the expression level of mRNAs by calculating fragments per kilobase of transcript per million fragments mapped (FPKM). Differentially expressed mRNAs were identified using the DESeq2 package, with the criteria of fold-change log2 > 1 or log2 < − 1 and with the statistical significance set to FDR < 0.05.

### Weighted gene correlation network analysis

WGCNA networks was generated for both age-related and tissue-related genes, following the overall approach described by Langfelder et al. [14]. Briefly, gene co-expression network was constructed based on a signed Spearman correlation; after network construction, modules are defined as clusters of densely interconnected genes by the topological overlap metric (TOM) and the dynamic tree cut algorithm [15] with a height of 0.25 and a deep split level of 2, a reassign threshold of 0.2, and a minimum module size of 30 (100 for the consensus network); module relationships were studied by eigenmodules–the first principal component of the module and a signature of gene expression, and each module that was correlated with the dose-response curve with a *p*-value < 0.01 was considered statistically significant.

## Supplementary information

**Additional file 1.**

**Additional file 2 Supplymentary Table 2**. List of DMRs among ages in breast tissue.

**Additional file 3 Supplymentary Table 3**. List of DMRs among ages in biceps brachii muscle tissue.

**Additional file 4 Supplymentary Table 4**. List of DMRs among ages in lung tissue.

**Additional file 5 Supplymentary Table 5**. WGCNA analysis of age-related genes.

**Additional file 6 Supplymentary Table 6**. WGCNA analysis of tissue-related genes.

## Data Availability

The DNA methylation data and RNA transcriptome data in this study are available in SRA database under the accession numbers PRJNA530286 and PRJNA512958, respectively. The yak reference genome is deposited in Dryad database (https://datadryad.org/stash/share/-99YgHwMBNaAWIo1TTw_BRclXHnpYq2ST4iZZpEE7bc).

## Abbreviations

QTP: Qinghai-Tibetan Plateau
DMRs: Differentially methylated regions
WGBS: Whole genome bisulfite sequencing
FPKM: Fragments per kilobase of transcript per million fragments
WGCNA: Weighted gene correlation network analysis
TOM: Topological overlap metric
GO: Gene ontology
KEGG: Kyoto encyclopedia of genes and genomes
A-DMRs: Age-related DMRs
ER: Endoplasmic reticulum
GS: Gene significance
MM: Module membership measures
